# Chronic unpredictable stress-induced inflammation and quantitative analysis of neurons of distinct brain regions in Wistar rat model of comorbid depression

**DOI:** 10.14202/vetworld.2020.1870-1874

**Published:** 2020-09-15

**Authors:** Vandana Blossom, Megha Gokul, Nayanatara Arun Kumar, Rekha D. Kini, Shyamala Nayak, K. Bhagyalakshmi

**Affiliations:** 1 Department of Anatomy, Kasturba Medical College, Mangalore, Manipal Academy of Higher Education, Manipal, Karnataka, India; 2 Department of Physiology, Kasturba Medical College, Mangalore, Manipal Academy of Higher Education, Manipal, Karnataka, India; 3 Department of Biochemistry, Kasturba Medical College, Mangalore, Manipal Academy of Higher Education, Manipal, Karnataka, India

**Keywords:** anxiety, C reactive protein, cortisol, depression, neuronal count, rat model, stress

## Abstract

**Background and Aim:**

Depression and anxiety are the most prominent neuropsychiatric disease and have been considered as the most burdensome diseases of society. The hippocampus and prefrontal cortex have a prominent role in stress-induced neurological disorders. Chronic unpredictable stress exposed rats are a perfect model in understanding comorbid depression and anxiety disorders. The inflammatory response occurring in the body has been linked to C-reactive protein (CRP) in many diseased conditions. The present research primarily focus on the possible correlation of Cortisol, CRP level and neuronal assay in different regions of hippocampus, dentate gyrus (DG), and prefrontal cortex.

**Materials and Methods:**

The control group of rats (n=6) was not exposed to any stress. Whereas, the experimental stress group (n=6) of rats was exposed to various stressors for 15 days. After the experimentation procedures, the blood samples were collected and brain dissection was done. The neurons in the prefrontal cortex, the DG along with various hippocampal regions was counted. Statistical analysis was performed using student’s t-test and p<0.05 was expressed as statistically significant.

**Results:**

Animals exposed to chronic unpredictable stressors showed a significant (p<0.0001) decrease in the neuronal count in prefrontal cortex and hippocampus. A significant rise in the serum cortisol (p<0.0001) and CRP (p<0.001) was witnessed in the stressed group.

**Conclusion:**

Our results demonstrate that chronic unpredictable stress exposure has affected neurogenesis in prefrontal cortex and hippocampal regions. Decreased neurogenesis was well in coordinance with the increase in cortisol and CRP. The chronic unpredictable stress-induced inflammatory response correlated to various brain regions might provoke insights into a variety of new drugs targeting neurogenesis.

## Introduction

Stress research has been documented as a prime concern in neurological research. Various regions of the brain have been involved in the regulation of the stress response. The brain regions are able to perceive and construe the stressors as potential threats [1]. During the exposure to stressors, fine-tuned functional neuroanatomical processing entails the involvement of distinct networks in brain. Various studies directing on the intricacy of stress are still challenging, but research in animals and humans have made substantial influences on its advancement [1]. Translational research has been helped in gaining significant progress in the field of stress integrating basic information and clinical practice. The stress response activates the hypothalamic-pituitary-adrenal axis and neuroinflammatory response prompting the generation of neuroinflammatory agents [2]. Stress is a risk factor for various psychological disorders, including depression and anxiety [1].

Animal models of depression depict the physiological disturbances that are homologous to different aspects of psychiatric disorders. Further, these models are proved to be helpful in understanding the comprehension of human psychopathology [3]. Research reports show the impact of chronic unpredictable stress on cellular integration and active involvement of certain brain regions such as DG, prefrontal cortex, and hippocampus [4,5]. The medial prefrontal cortex is an essential neural substrate helping in modulating the hypothalamic-pituitary-adrenal axis during the exposure to stressful situations [6].

Neuronal plasticity has been effected in various target regions of the brain during repeated stressful experiences [6]. Multiple studies have shown that stress-induced neuroinflammation is accompanied by increased production of oxygen radicals producing adverse effects in the hippocampus causing neuronal death. Hippocampus and prefrontal cortex are easily vulnerable to chronic stress exposure leading to various neurological problems [7]. C reactive protein (CRP) has been linked to inflammation and tissue destruction [8]. High CRP concentration has been associated with cognitive impairment and neurodegenerative disorders. Decreased number of pyramidal cells in hippocampus has been reported in various experimental stress experiments. Chronic stress has also been shown to augment the noradrenergic innervation in the prefrontal cortex.

The present study was aimed to correlate the effect of chronic unpredictable stress on CRP, cortisol, and neuronal cell count in Wistar rat model of comorbid depression.

## Materials and Methods

### Ethical approval

All the procedures for the animal experiments were reviewed and approved by the Institutional Animal Ethical (IAEC) at Kasturba Medical College, Mangalore, on January 27, 2018 (KMC/MNG/IAEC/03-2018). All animal studies were conducted and maintained according to guidelines proposed by the Committee for Control and supervision of experimentation on animals (CPCSEA), Government of India.

### Study period and location

This study was conducted at Kasturba Medical College, Mangalore during the period from January 2020 to February 2020.

### Animals used for the experimentation procedure

Adult Wistar albino rats weighing 160-200 g were used for the present experimentation procedures. The animals were obtained from the Central Animal House Kasturba Medical College, Mangalore. Animals were housed individually in polypropylene cages (29 cm×22 cm ×14 cm) with paddy husk bedding. Throughout the research period, all the animals were maintained at 25±2°C temperature with 12 h of light and dark cycles. Further, rats were provided with *ad libitum* access to laboratory food (commercial rat pellets from VRK nutritional solutions, India) and water.

### Chronic unpredictable stress procedure

The rat model of comorbid depression involved the exposure of chronic unpredictable stressors. In this experiment, the animals were exposed to unpredictable stressors for 15 consecutive days. This model was devised to exaggerate the unpredictability [3,4]. The animals were exposed to stress each day, at different times to lessen predictability and avoiding habituation. The procedures were done in a quarantined room adjacent to the rat housing for minimal handling or transport of the rats. Soon after each stressor, all the rats were kept in the recovery room for 1 h following which they were shifted to their original rooms. Restraint stress involved the placing of rats in a restraining device made of Plexiglas and flexible nylon for 1 h. During restraint, all the movements of animals were restrained but there was access to respiration and air circulation. In the rotation procedure, the rats were placed on the rotating spinner (50 rpm) for 1 h. Cage wetting stress was given by keeping rats in a cage with wet husk (5 cm high) for 4 h. When the rats were subjected to swimming stress (Warm and cold), the rats were allowed to swim in a cylindrical tank (60 cm height × 30 cm diameter) filled with water to a 30 cm depth at 45 and 8°C, respectively. Footshock (1.5 mA) was given through the grid floor of a chamber enclosed within a skinner box (30 s on, 120 s off, for 10min). Placing the rats in the dark in the day time and light in night time influenced the light and dark cycle. Tail pinch involved be placing a clothes pin at 2 cm from the base of the tail for 20 min. Isolation stress was given by placing individual rats in the different home cage (4 h). Overcrowding stress was done by placing six to seven rats in a single small cage for 2 h. Cage tilting involved the placement of rats in the cage and tilting at an angle of 45°. Overnight food deprivation and overnight water deprivation were also involved as a procedure of stress. In the heat stress, the rats were exposed to a hot air stream from a hairdryer for about 20 min. Cold stress will be given by exposing the rats in the cold chamber at 8°C for 1 h. The entire sequence of chronic unpredictable stressors is represented as follows.

### Experimental schedule for the chronic unpredictable stress model

**Table:** 

Day 1	1 h restraint
Day 2	1 h rotation
Day 3	4 h of wet bedding
Day 4	30-min warm water swim (45°C)
Day 5	10-min of mild shock
Day 6	20-min cold water swim (8°C)
Day 7	4 h of isolation
Day 8	20-min tail pinch in the restrainer
Day 9	Inversion of light and dark cycle
Day 10	2 h Overcrowding stress
Day 11	Cage tilting for 60 min
Day 12	Food deprivation overnight
Day 13	Hot air stream for 20 min
Day 14	Water deprivation overnight
Day 15	Cold stress

At the end of the 15^th^ day, the animals were anesthetized with a combination of ketamine and xylazine (50 mg/mL of ketamine and 20 mg/mL of xylazine). Cardiac puncture was done, and the blood samples were collected in the vacutainers for the estimation of cortisol [9], and CRP was estimated by standard kit methods [10].

### Neuronal assay of frontal cortex and hippocampus [11]

After anesthetization, the animals were placed on the dissection board. The chest cavity was opened to expose the heart. The perfusion is done using normal saline (0.9%) about 150 mL, through the left ventricles, which is then followed by 10% formalin. Brain tissue was removed and stored in 10% formalin for 48 h for the process of fixation. Once the fixation has been done, the paraffin blocks were made. Coronal sections of the frontal cortex and hippocampus were made using a rotary microtome. All the prepared were stained with cresyl violet stain.

### Quantification of neurons

The quantitative analysis of the neurons was performed under light microscopy (20×). In the frontal cortex section, the neurons were counted in 300×300 μ areas. In the dentate gyrus (DG), 150×150 μ area was selected for counting. Hippocampus area cornu ammonis (CA) 1, CA2, CA3, and CA4) were also selected for quantifications. Neurons were quantified using imaging software NIS Elements.

### Statistical analysis

The obtained results are expressed as Mean ± SD. The differences between groups were compared for statistical significance by Student t-test, with the level of significance set at p < 0.05.

## Results

**Figure-1:**
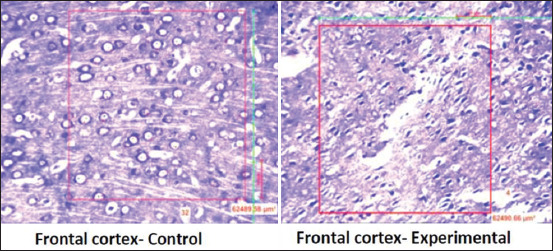
Distribution of neuronal count in the frontal cortex in the control group and experimental group.

**Figure-2:**
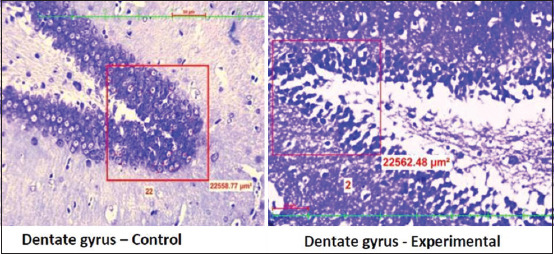
Distribution of neuronal count in the dentate gyrus in control group and experimental group.

**Figure-3:**
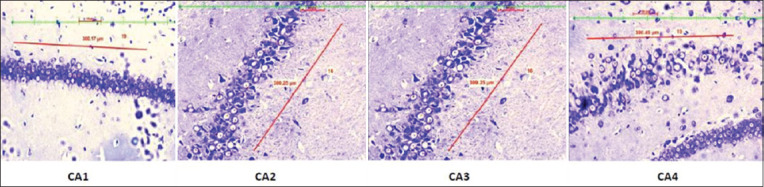
Distribution of neuronal count in different Hippocampus regions (CA1, CA2, CA3, and CA4) in the control group.

**Figure-4:**
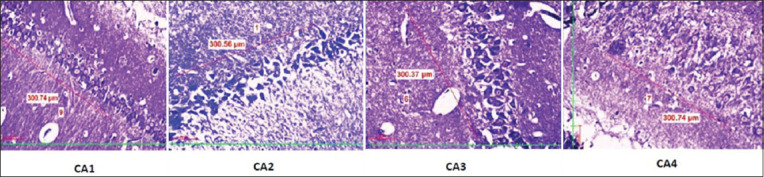
Distribution of neuronal count in different hippocampus regions (CA1, CA2, CA3, and CA4) in the experimental group.

A significant (p<0.0001) rise in the serum cortisol level and CRP level in the stressed group was observed in the stress group when compared to the control group (Table-1). Further, neuronal count (Table-2) significantly (p<0.0001) declined in the prefrontal cortex (Figure-1) DG (Figure-2) and CA 1, CA2, CA3, and CA4 regions of the hippocampus in the experimental group of rats when compared to the control group (Figures-3 and 4).

**Table-1:** Effect of chronic unpredictable stress on C reactive protein and serum cortisol.

Parameters	Control group (n=6)	Experimental group (n=6)
C reactive protein (mg/L)	0.183±0.009	0.375±0.04***
Cortisol (µg/dL)	2.40±0.04	8.32±0.42***

p<0.001; control versus experimental group

**Table-2:** Effect of chronic unpredictable stress on quantitative analysis of the neurons in different brain region.

Brain regions	Control group (n=6)	Experimental group (n=6)
Frontal cortex	29±0.89	7±0.63***
Dentate gyrus	41.5±0.31	4±0.63***
CA4	21.5±0.83	3±1.67***
CA3	21.5±1.04	2±0.632***
CA2	33±0.89	8±0.632***
CA1	26.5±0.54	6±0.63***

p<0.001; control versus experimental group

## Discussion

Depression and anxiety were the most prevailing psychiatric disorder in the present society [12]. Cell death of the neurons is one of the potential outcomes of oxidative stress. Chronic stress badly effects most of the physiological variations in the central nervous system contributing to the degeneration of neurons. One of the most susceptible regions is the limbic system which is associated with the regulation of cognitive and behavioral functions [13]. Depression augments the inflammatory reactions and intensifies the increased formation of pro-inflammatory agents leading to the pathological process. In the present study, serum cortisol levels increased in the rats exposed to chronic unpredictable stressors. Corticotropin-releasing factor has a potent role in the stress response by modulating the hypothalamic-pituitary-adrenal axis. During the exposure to stress, the corticotropin-releasing factor causes the initiation of events that augments the release of glucocorticoids from the adrenal cortex. The stress-induced increase in the serum cortisol observed, in this study, is in accordance with the previous study reports [14,15].

Stress-induced morphological changes have been well associated with the hippocampus. Adult neurogenesis has been linked to the functional components of the hippocampus. In the present study, neuronal count in DG, prefrontal cortex, and hippocampus regions (CA1, CA2, CA3, and CA4) decreased drastically in the animals exposed to chronic unpredictable stressors. The brain imaging studies in post-traumatic stress disorder patients reported a decrease in the hippocampal volume associated with memory deficit [16]. Exposure to immobilization stress also reduced the dendritic spine and branches of pyramidal neurons in the CA3 region of the hippocampus [17]. Further, restraint stress-induced alterations in the production and survival of new neurons in the hippocampus have also been reported [18]. The prefrontal cortex is vulnerable to the deleterious effects of stress and has been well associated with the regulatory influence of stress on the hippocampus [19]. The medial part of the prefrontal cortex through its diverse connections to the amygdala and hippocampus plays an active role during stress responses [20]. The decreased neuronal count observed in our study, is in accordance with the previous study reports [16-20].

Stress could be the prime cause for the augmentation of the noradrenergic innervation. The increased level of CRP observed in this study illustrates the inflammatory response during the stress. Our previous research work depicted the rise in liver enzymes and lipid peroxidation level with the decrease in the antioxidant level during the exposure to chronic unpredictable stressors. Moreover, all these parameters were positively correlated with an increase in the serum cortisol and CRP [21]. In this study, we correlated the alteration of inflammatory marker CRP with the neuronal variation in distinct brain regions. The observed neuronal variation considered in this study is proved to be the target region in various neurodegenerative diseases. A Positive association was observed between the increased CRP and decreased neuronal count in frontal cortex, DG, and various regions of the hippocampus. Elevated stress hormones might be initiating an inflammatory response involving releasing inflammatory mediators in the brain tissues. Decreased neurogenesis was well associated with the increase in cortisol and CRP. The stress hormone and the inflammatory marker are believed to affect the neurogenesis. The present results suggest that chronic unpredictable stress might induce inflammatory responses causing diverse neuroinflammation associated with psychiatric diseases.

## Conclusion

The chronic unpredictable stress model chosen in this study is a potent model of depression and anxiety, preventing habituation and activating the inflammatory mechanisms as documented in this study. CRP could be the prime biomarker of inflammation implicating the pathophysiological aspects of stress-induced neuronal damage. Understanding the molecular mechanisms triggered in response to stress is very useful for the advancement of pharmacological innovations, which could be used in the treatment of diseases caused by stress. Further in-depth research is necessary to evaluate the possible effects of stress-induced neurodegeneration and its progression. Inflammatory markers should be considered as the potent biomarker in neuropsychiatric disorders which could avoid the progression of subclinical brain damage. The chronic unpredictable stress-induced inflammatory response might provoke an insight into a variety of new drugs targeting to the management of stress-induced anxiety and depression.

## Authors’ Contributions

NAK: Conceptualization, drafted and reviewed the manuscript. VB: Histological sectioning and neuronal count. MG and RDK: Performed experimental procedures in rats. SN and KB: Performed the data acquisition and statistical analysis. All authors read and approved the final manuscript.
